# Minimizing the threat of pandemic emergence from avian influenza in poultry systems

**DOI:** 10.1186/1471-2334-13-592

**Published:** 2013-12-16

**Authors:** Kim M Pepin, James O Lloyd-Smith, Colleen T Webb, Karen Holcomb, Huachen Zhu, Yi Guan, Steven Riley

**Affiliations:** 1 Research and Policy in Disease Dynamics Program, Fogarty International Center, Bethesda, MD, USA; 2 Department of Biology, Colorado State University, Fort Collins, CO, USA; 3 Department of Ecology and Evolutionary Biology, University of California, Los Angeles, Los Angeles, CA, USA; 4 State Key Laboratory of Emerging Infectious Diseases, The University of Hong Kong, Pokfulam, SAR, PRC Hong Kong; 5 MRC Centre for Outbreak Analysis and Disease Modelling, Department of Infectious Disease Epidemiology, School of Public Health, Imperial College London, London, UK

**Keywords:** Avian influenza, Live-bird markets, Efficacy of controls, Environmental transmission, Wholesale markets

## Abstract

**Background:**

Live-animal markets are a culturally important feature of meat distribution chains in many populations, yet they provide an opportunity for the maintenance and transmission of potentially emergent zoonotic pathogens. The ongoing human outbreak of avian H7N9 in China highlights the need for increased surveillance and control in these live-bird markets (LBMs).

**Discussion:**

Closure of retail markets in affected areas rapidly decreased human cases to rare, sporadic occurrence, but little attention has been paid thus far to the role of upstream elements of the poultry distribution chain such as wholesale markets. This could partly explain why transmission in poultry populations has not been eliminated more broadly. We present surveillance data from both wholesale live-bird markets (wLBMs) and rLBMs in Shantou, China (from 2004–2006), and call on disease-dynamic theory to illustrate why closing rLBMs has only minor effects on the overall volume of transmission. We show that the length of time birds stay in rLBMs can severely limit transmission there, but that the system-wide effect may be reduced substantially by high levels of transmission upstream of retail markets.

**Summary:**

Management plans that minimize transmission throughout the entire poultry supply chain are essential for minimizing exposure to the public. These include reducing stay-time of birds in markets to 1 day, standardizing poultry supply chains to limit transmission in pre-retail settings, and monitoring strains with epidemiological traits that pose a high risk of emergence. These actions will further limit human exposure to extant viruses and reduce the likelihood of the emergence of novel strains by decreasing the overall volume of transmission.

## Background and discussion

Retail live-bird markets (rLBMs) LBMs are the main focus of control of AIVs in poultry supply chains because this is where the largest number of humans contact live birds, and humans have been infected repeatedly with strains circulating in rLBMs [1,2]. Retail LBMs are thought to provide a mixing ground for the emergence of novel strains because strains from different sources are brought together in a setting with high host-species diversity and density [3]. However, this hypothesis requires that transmission followed by viral replication and possibly reassortment occurs within rLBMs. Perhaps surprisingly, little is known about how rLBMs contribute to AIV transmission. A recent analysis of surveillance data from rLBMs in Shantou, China found that the prevalence of H5 and H9 in chickens could not be predicted from their corresponding prevalences in ducks and quail within those same markets [4], suggesting that a substantial proportion of transmission may occur in contexts other than rLBMs. Also, AIVs can be isolated at high rates from other holdings that form the supply chain for rLBMs [5].

### Insight from basic epidemic theory

Despite a lack of empirical studies of the transmission of influenza in rLBMs, well-established ideas from epidemic theory enable us to make mechanistic predictions about prevalence patterns within them. Incubation periods for AIVs in poultry can be up to 2 days when birds are inoculated with doses less than or equal to 10^3^, and around 1 day when doses are higher [6]. Making the worst-case assumption that susceptible and infectious hosts are in constant contact, this means that the minimum time infectious individuals can create other infectious individuals is 1 day, and higher on average. Thus, if the average stay-time of birds in rLBMs is ≤ 2 days, there is not time for exponential growth of prevalence due to direct transmission within rLBMs (e.g., “outbreaks”). In addition, direct transmission alone may not cause significant amplification of prevalence within rLBMs because birds that entered the market uninfected have a high probability of being slaughtered before they begin shedding AIV. A similar principle has been identified in other animal-disease systems. For example, epizootics of plague in prairie dog populations have been shown not to occur by blocked-flea or pneumonic transmission alone, because both blocked vectors and hosts capable of direct transmission are removed from the population by death before they reliably create large chains of transmission required for outbreaks [7]. Essentially, direct transmission in rLBMs should be limited by the interplay of stay-times and incubation periods.

Retail LBMs are also thought to foster persistence of AIVs in the environment, creating another source of transmission [3]. The importance of an environmental factor in viral persistence has been shown in an experiment that monitored AIV isolation rates before and after days that the market was disinfected [8]. However, whether this environmental persistence adds to transmission has not been determined empirically. Theoretically, indirect transmission via an environmental reservoir could contribute to the overall force of infection within rLBMs by providing a sustained source of AIV (i.e., by providing a transmission link between birds even if they do not occupy the market at the same time) [9]. Indirect transmission can occur through a variety of routes, including viruses in drinking water, in feces on the ground or on surfaces in cages. All of these routes rely on three main processes: shedding rates into/on a particular environmental feature, decay rates of the virus in it, and contact of susceptible birds with it. Intuitively, one would predict that indirect transmission would be most significant when shedding rates are high, decay rates are low and contact rates are high.

### Models can illustrate the amplitude of these trade-offs

**Figure 1:**
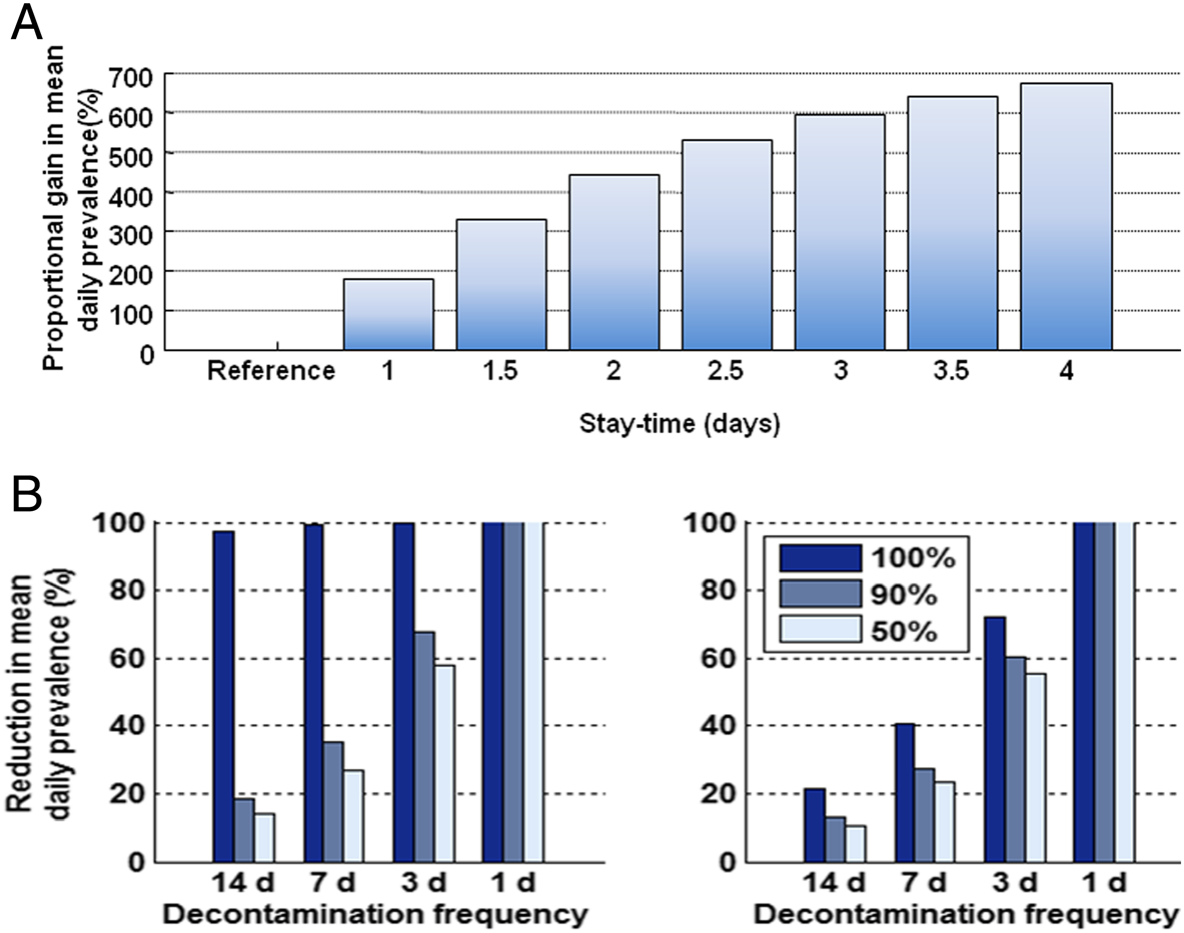
**Model output. A**. Effects of stay-time in retail markets on infection prevalence. Impact of stay-times on prevalence is most dramatic at stay-times less than 2 days. Simulations (N = 3528; for all possible combinations of parameters in Additional file 1: Table S1) were run under different combinations of parameter values (see Additional file 2: Figure S1) for each stay-time indicated on X-axis. Mean daily prevalence (over 1 year) was calculated for each parameter set. Each bar reflects an overall mean across parameter sets. These overall means were expressed as prevalence relative to the lowest stay-time (0.5 days; i.e., bars show ((mi – m0.5) / m0.5)×100, where m = mean prevalence across parameters sets, i = 1, 1.5, 2.5, 3, 3.5, 4 days). Absolute values of prevalence will depend on specific parameter values; qualitative results are robust; higher transmission rates lead to larger effects of stay-time (Additional file 2: Figure S1B). **B**. Impact of influx of chickens on effectiveness of decontamination in retail markets. Difference between mean prevalence of the ‘reference’ scenario (no disinfection routine) and mean prevalence under each treatment, divided by the reference and expressed as a percent. The frequency of disinfection is shown on X-axis. Efficacy of disinfection (i.e. 100, 90 and 50%) is indicated in the legend with a stay-time of 3 days. Left panel: Infected hosts were introduced only once at the start of the simulation (initial prevalence = 0.1%) and only susceptible or recovered hosts entered the market thereafter. Right panel: The number of infected hosts entering retail markets at each time step was chosen randomly from the distribution of prevalences in the wholesale market (Figure 2, red bars).

We illustrate the tradeoffs between these three processes using a simple mathematical model (Additional file 1: Text S1). Contact rates with contaminated environmental reservoirs and shedding rates are the strongest determinants of mean daily prevalence in poultry in rLBMs (Additional file 2: Figure S1). Our simple model also helps to illustrate how transmission levels within rLBMs are strongly dependent on stay-times in markets. When the incubation period of an emergent virus in a susceptible host is 1 day, mean daily prevalence increases by 250% when stay-time is increased from 1–2 days (Figure 1A). The rise in prevalence is not linear: it slows for stay-times greater than 3 days. The remarkable effects of stay-time on prevalence highlights why bans on overnight poultry storage in live-bird markets in Hong Kong have been so effective at eliminating AIVs [10]. Furthermore, although high-demand poultry such as chickens tend to have short stay-times in markets, quail and other minor poultry may remain longer due to lower demand. The longer stay-times for quail could partly explain why they have been implicated as intermediate hosts in the emergence of novel strains, and why excluding them from rLBMs in Hong Kong is associated with reduced prevalence in chickens [11]. Short, standardized stay-times for all host species in markets seems important for both controlling AIV prevalence and, potentially, for gaining a fundamental understanding of which host species may contribute most to the risk of emergence in humans.

We used the model to investigate the relationship between: incidence of infection in rLBMs; the use of decontamination practices (that decrease environmental transmission); and the rate of influx of susceptible birds. When decontamination routines remove 100% of virus from the environment and there are no infected birds entering retail markets from wholesale markets, as would be expected, decontamination is > 99% effective even when it occurs infrequently (i.e., every 2 weeks) (Figure 1B, left). In contrast, when continual introduction of AIV from wLBMs contributes to prevalence levels in rLBMs, as is likely the case, rigorous decontamination (100% efficacy) is only 50% effective at decreasing environmental contamination when decontamination occurs as often as weekly (Figure 1B, right). The constant influx of infected birds leads to conditions where decontamination must occur more often than weekly in order to reduce environmental contamination by more than 50% Figure 1B, right). However, when decontamination practices are less than 100% effective, prevalence of the virus in the environment is much less affected by the influx of susceptible birds (Figure 1B) and stay-times (Additional file 3: Figure S2) showing that there is less opportunity for the management of stay-times to act synergistically with decontamination (Figure 1B and Additional file 3: Figure S2). In order to design effective strategies to eliminate AIVs from rLBMs, there is a need for systematic empirical studies to accurately quantify the role of prevalence of infection in upstream components of the poultry production system (e.g., wLBMs, farms and intermediate holdings) in reducing the efficacy of decontamination practices at rLBMs.

**Figure 2:**
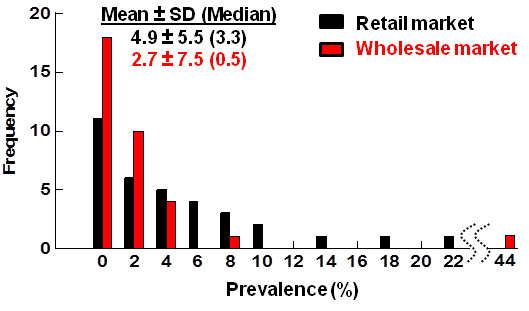
**Distribution of bi-weekly prevalence samples of H9 in chickens between 2005–2006 in Shantou, China in the largest wholesale market (red) and nine retail markets (black).** Each monthly estimate of prevalence was considered. The x-axis represents monthly prevalence of H9 binned at an interval of 2% prevalence. Note that one monthly sample from the wholesale market was unusually high at 44% and thus the plot is truncated between 22 and 44% as indicated by the squiggly dotted lines. The means ± 1 standard deviation (and medians enclosed in brackets) for the monthly prevalence measures indicate that prevalence in wholesale markets may be as much as 50% that in retail markets.

**Figure 3:**
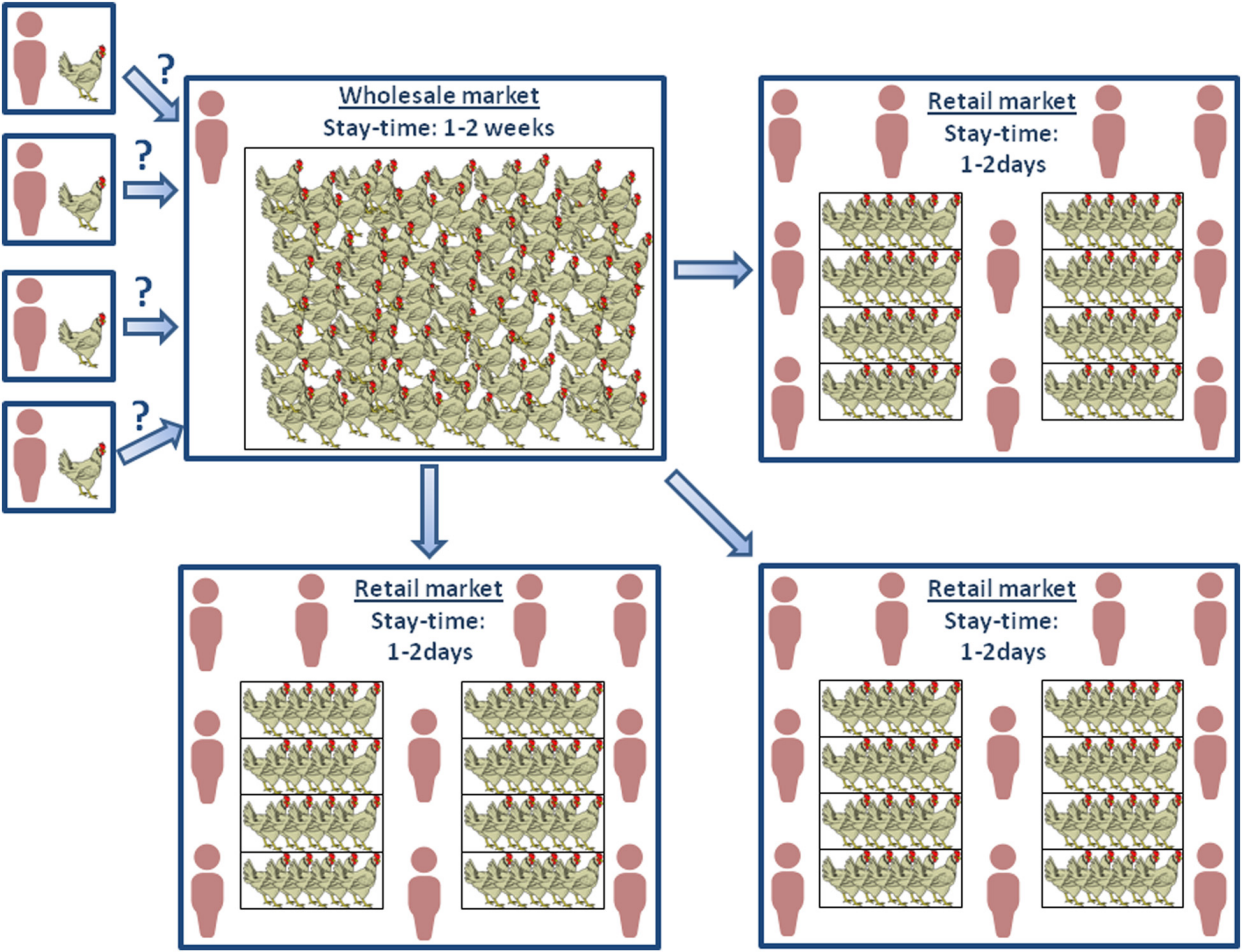
**Schematic of the poultry supply chain**. Birds are brought to wholesale markets from several independent (often undocumented) locations and flock owners. The source for birds changes constantly and varies from large-scale operations with thousands of birds per farm to family-owned backyard flocks. In wholesale markets, birds are kept at very high density in large pens. There is interaction with poultry caretakers as well as poultry traders, but not the general public. From wholesale markets, birds are brought to several retail markets where they are kept at high density in small, stacked cages. Thus, the bird population is more structured in retail markets. Also, the stay-time of birds in retail markets is much shorter than in wholesale markets.

The model results are calibrated with surveillance data for H9 subtypes in a major wLBM and nine rLBMs in Shantou, China [5], showing that average prevalence in rLBM is roughly twice as high as prevalence in wLBM (means ± standard deviation (medians): 4.9% ± 5.5% (3.3%) in rLBM versus 2.7% ± 7.5% (0.5%) in wLBMs; Figure 2). Comparison of these numbers alone does not reveal how much transmission occurs in either setting because birds could have been infected prior to arrival in either setting [12]. For example, some birds may become exposed in wLBMs but are not detected as infected until they enter rLBMs. Combining this logic with the observed prevalence data suggests that > 50% of the transmission that created this prevalence in rLBMs occurs before the birds arrive. Conditions in wLBMs may create potent opportunities for transmission: birds in wLBMs are kept at high density in one space (rather than separated in cages as in rLBMs) and birds remain longer in wLBMs than rLBMs (Figure 3).

### Going beyond H5N1

The scientific community forecasted key elements of the emergent H7N9 virus by prior surveillance, laboratory studies and clinical observations [13,14]. H9N2 viruses were predicted to pose a risk of novel-strain emergence due to their: propensity to co-infect with other subtypes, dominance in rLBMs, broad host range, dominance in chickens (the most numerous poultry species), low virulence (i.e., low detectability) and capability of infecting humans [5,13-15]. These warnings were first publicized 14 years ago when it was suggested that genetic material from H9N2 subtypes contributed to the emergence of H5N1 in 1997 in Hong Kong [16]. The recent finding that the internal genes of the novel H7N9 strain come from an H9N2 strain [2] validates predictions from surveillance data and further emphasizes the need to understand the multi-host epidemiology of H9N2 viruses. Prevention of the emergence of novel strains with pandemic potential will require the identification of strains that are high risk such as H9N2 and the taking of measures to eliminate them. Once elimination is achieved, it may be appropriate to list these strains under the World Organization for Animal Health (OIE) definition for high-risk strains so that more extreme measures can be used to control their re-introduction. In any case, it is worth placing more emphasis on control of emergent strains - those with epidemiological traits that favor emergence - not just those that have shown a history of causing HP AIV infections.

## Summary

Our model demonstrates how the interplay between stay-time of birds in rLBMs, prevalence of infection in birds entering the rLBMs, and levels of infectious virus within rLBMs ultimately determine how effective a specific intervention will be. Combined with surveillance data from Shantou, several general guidelines for surveillance and control of AIV in poultry become clear. In rLBMs, the lowest risk to humans will be achieved by limiting stay-times to 1 day for all bird species, conducting effective decontamination as frequently as possible and taking measures to minimize the persistence of AIVs in drinking water, such as altering water temperature, pH, salinity or organic content [9,17]. This has been achieved in Hong Kong and thus should be possible more broadly. However, AIVs will continue to threaten public health until their prevalence is reduced in other holdings that supply rLBMs. Due to the complex patterns of host species usage by different AIV subtypes [5], other important steps to decrease transmission (and potential reassortment) in wLBMs and other pre-rLBM settings include reducing stay-time, implementing structures to minimize host mixing, and preventing cross-species contact of shared resources.

Repeated outbreaks of avian influenza in humans may eventually justify more substantial changes to the poultry distribution system in some countries. A broad restructuring of the poultry distribution system would greatly help to reduce environmental contamination and thus risk of transmission in both poultry and humans. Key changes would include a shift from rLBMs to cold-chain distribution, and introduction of a centralized slaughter system with standardized, hygienic practices for slaughter, decontamination, and waste processing. These changes are more difficult to implement in some countries because of the cultural and social importance of rLBMs as well as the ever-changing animal-trade network which involves many small businesses. Nevertheless, policies that require standardized poultry supply activities, with minimal stay-times of birds in any specific holding and minimal transfers between holdings before entering rLBMs are essential to control the transmission of AIVs in poultry populations, and thus prevent the emergence of novel strains. Systematic sampling and testing in a geographically representative sample of large Chinese wLBMs should be undertaken urgently. Both the 2009 emergence of human H1N1 in the North American pork supply chain and the 2013 H7N9 outbreak of H7N9 in the Chinese poultry supply chain motivate a transition from ad hoc academic studies to systematic representative surveillance of influenza A viruses in key livestock populations.

## Competing interests

The authors declare that they have no competing interests.

## Authors’ contributions

Conception and design: KP, SR, JLS, CW; Analyses: KP, KH; Data collection and sampling design: HZ, YG; Wrote the manuscript: KP; Edited the manuscript: SR, JLS, CW; All authors read and approved the manuscript.

## Pre-publication history

The pre-publication history for this paper can be accessed here:

http://www.biomedcentral.com/1471-2334/13/592/prepub

## Supplementary Material

Additional file 1 Supplementary materials. Click here for file

Additional file 2: Figure S1 **A.** Uncertainty analysis for transmission parameters. Set of mean daily prevalence over 1 year for different stay-times in retail markets (x-axis). Results are from all possible combinations of the parameter values listed in Additional file 1: Table S1 for transmission rate, shedding rate, decay rate and proportion susceptible. Ten infected hosts (0.1% of N) were introduced once on day 0. Medians for each set of parameters are indicated as small circles. Results within the 75th quantile include the thin lines and below them. **B.** Effects of individual parameters on model output. Each point represents a simulation with the x-axis value of the indicated parameter and all possible values (given in A) for each other parameter. PCC indicates partial correlation coefficients for mean daily prevalence and the value of a particular parameter. This shows the effects of a particular parameter regardless of all other parameters. For example, transmission and shedding rates have the strongest effects on mean daily prevalence, where higher rates mean higher prevalence. Click here for file

Additional file 3: Figure S2 Same as Figure 1B except that stay-time was 1 day. Click here for file
