# Supplementary material for: Minimizing the threat of pandemic emergence from avian influenza in poultry systems
Source: BMC Infect Dis. 2013 Dec 16;13:592. doi: 10.1186/1471-2334-13-592 (PMC3878446; doi:10.1186/1471-2334-13-592)
Supplement: Additional file 1 — Supplementary materials. [file 1471-2334-13-592-S1.docx]

**Supplementary materials**

**Methods:** Text S1.

*Model*

We used the simplest model structure that is consistent with basic mechanisms of transmission in live-bird market settings, in order to explore the dynamics in a parsimonious manner. This structure consisted of a system of ordinary differential equations which describe the infection dynamics of a single strain of AIV in a single host species in a retail live-bird market. Host compartments include susceptible (*S*), Exposed (*E*), Infectious (*I*) and Removed (*R*). A fifth compartment describes the concentration of viruses in the environment (W). The force of infection (ϕ) is determined solely by contact rates with the contaminated environment, restricted by a half-saturation constant (the minimum dose at which 50% of susceptible individuals become infected) [1].

λ = β(*W/(W+k*) (1)

The number of *S,* *E*, *I*, and *R*, birds and the concentration of infectious agents in the environment, *W* are given by:

dS/dt = p_S_Nμ - Sμ - λS (2)

dE/dt = - Eμ + λS - Eε (3)

dI/dt = p_I_Nμ - Iμ + Eε - Iγ (4)

dR/dt = p_R_Nμ - Rμ + Iγ (5)

dW/dt = σI - δW (6)

where p_i_ indicates the proportion of birds in each disease state *i* that enter retail markets, 1/μ is the number of days birds stay in retail markets before being sold (“stay-time”), 1/ε is the incubation period in days, 1/γ is the infectious period in days, σ gives the rate at which viral particles are shed into the environment and δ is the rate at which viral particles decay in the environment (Table 1)*.* We assumed that the host population in retail markets was constant (i.e., *S + E + I + R = N*) to match the constant supply/demand structure of retail markets. We allowed for extinction of infectious individuals in simulations by setting the number of infected hosts to 0 when the estimated number dropped below 1. Similar results were obtained using a stochastic version of this model.

In order to understand the impact of supply populations on the efficacy of disinfection routines, we compared two methods for introducing infected hosts: 1) 10 infected hosts (0.1% of population) introduced once at the start of simulations, or 2) a random daily input based on a probability distribution of the prevalence in a major wholesale market (implemented through the p_I_ term at time t). We modeled cleaning routines by removing all hosts and decreasing the concentration of viruses in the environment either by a fixed proportion. The population of S, E, I and R was restocked according to their respective proportions at time t. In order to introduce decontamination on specific days, we ran the model by solving the system of equations for one day at a time.

We ran all simulations for 1 year , using a large range of parameter values (Table S1) for parameters that are not well specified by empirical research. A full-factorial sampling of the values for variable parameters listed in Table 1 was conducted. We fixed incubation periods at 1 day, which is the most conservative estimate for our analysis (i.e., incubation periods can be 2 days or more [2] depending on viral strain and dose, which would lead to even lower transmission levels in markets). We fixed incubation periods at 7 days because experiments have found an average value of 6-7 days for chickens, and longer infectious periods would be more conservative for our analyses [3]. We assumed that no exposed hosts entered the markets, so that we could control the number of incoming hosts that were infectious using the p_I_ parameter. We show the variability produced by the ranges of parameter values in Figure S1A and relationships for specific parameters and prevalence in Figure S1B. To produce Figures 1 and 2 in the main text, we used the median values from the set of all parameters.

**References**

1. Rohani P, Breban R, Stallknecht DE, Drake JM: **Environmental transmission of low pathogenicity avian influenza viruses and its implications for pathogen invasion.** *Proc Natl Acad Sci U S A* 2009, **106:** 10365-10369.

2. Spickler AR, Trampel DW, Roth J: **The onset of virus shedding and clinical signs in chickens infected with high-pathogenicity and low-pathogenicity avian influenza viruses.** *Avian Pathol* 2008, **37:** 555-577.

3. van der Goot JA, Koch G, de Jong MCM, van Boven M: **Quantification of the effect of vaccination on transmission of avian influenza (H7N7) in chickens.** *Proc Natl Acad Sci U S A* 2005, **102:** 18141-18146.

4. Brown JD, Poulson R, Carter DL, Lebarbenchon C, Stallknecht DE: **Infectivity of avian influenza virus-positive field samples for mallards: what do our diagnostic results mean?** *J Wildl Dis* 2013, **49:** 180-185.

5. Pantin-Jackwood MJ, Swayne DE: **Pathogenesis and pathobiology of avian influenza virus infection in birds.** *Rev Sci Tech* 2009, **28:** 113-136.

6. Leung YHC, Zhang LJ, Chow CK, Tsang CL, Ng CF, Wong CK, Guan Y, Peiris JSM: **Poultry drinking water used for avian influenza surveillance.** *Emerg Infect Dis* 2007, **13:** 1380-1382.

**Table S1.** Model parameters.

| **Parameter** | **Biological Description** | **Values (range)** |
| --- | --- | --- |
| *N* | Host population into the retail market | 10^4^ hosts |
| *p_S_* | Proportion of incoming birds that are susceptible | 10, 20, 30, 40, 50, 60, 70, 80, 90 % of hosts |
| *p_I_* | Proportion of incoming birds that are infectious | 0.1% once or prevalence values from wholesale data |
| *p_R_* | Proportion of incoming birds that are recovered | 1-(p_S_ + p_I_) % of hosts |
| 1/μ | Length of stay in the retail markets | 0.5, 1, 1.5, 2, 2.5, 3, 3.5, 4 days |
| β | Contact rate with contaminated environment | 0.001, 0.005, 0.01, 0.05, 0.1, 0.5, 1 day^-1^ |
| 1/ε | Incubation period | 1 day [2] |
| 1/γ | Infectious period | 7 days [3] |
| *k* | Minimum dose that results in 50% of the maximum proportion of infections | 10 particles [4,5] |
| σ | Rate of shedding of viral particles into the environment | 0.001, 0.005, 0.01, 0.05, 0.1, 0.5, 1 particles * ml^-1^ * days^-1^ |
| *δ* | Decay rate of viral particles in the environment | 0.25, 0.35, 0.45, 0.55, 0.65, 0.75, 0.85, 0.95 particles * ml^-1^ * days^-1^ [6] |

**Supplementary Figure Legends**

**Figure S1. A.** Uncertainty analysis for transmission parameters. Set of mean daily prevalence over 1 year for different stay-times in retail markets (x-axis). Results are from all possible combinations of the parameter values listed in Table S1 for transmission rate, shedding rate, decay rate and proportion susceptible. Ten infected hosts (0.1% of N) were introduced once on day 0. Medians for each set of parameters are indicated as small circles. Results within the 75th quantile include the thin lines and below them. **B.** Effects of individual parameters on model output. Each point represents a simulation with the x-axis value of the indicated parameter and all possible values (given in A) for each other parameter. PCC indicates partial correlation coefficients for mean daily prevalence and the value of a particular parameter. This shows the effects of a particular parameter regardless of all other parameters. For example, transmission and shedding rates have the strongest effects on mean daily prevalence, where higher rates mean higher prevalence.

**Figure S2**. Same as Figure 1B except that stay-time was 1 day.
