# Supplementary figures and images for: Minimizing the threat of pandemic emergence from avian influenza in poultry systems
Source: BMC Infect Dis. 2013 Dec 16;13:592. doi: 10.1186/1471-2334-13-592 (PMC3878446; doi:10.1186/1471-2334-13-592)

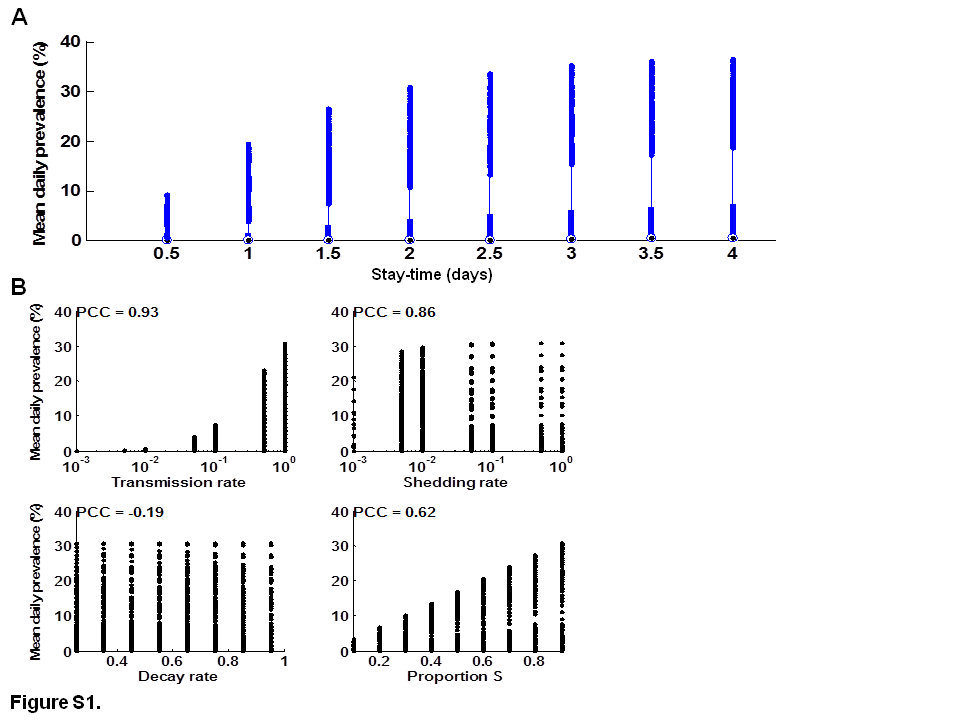

Supplement: Additional file 2: Figure S1 — A. Uncertainty analysis for transmission parameters. Set of mean daily prevalence over 1 year for different stay-times in retail markets (x-axis). Results are from all possible combinations of the parameter values listed in Additional file 1: Table S1 for transmission rate, shedding rate, decay rate and proportion susceptible. Ten infected hosts (0.1% of N) were introduced once on day 0. Medians for each set of parameters are indicated as small circles. Results within the 75th quantile include the thin lines and below them. B. Effects of individual parameters on model output. Each point represents a simulation with the x-axis value of the indicated parameter and all possible values (given in A) for each other parameter. PCC indicates partial correlation coefficients for mean daily prevalence and the value of a particular parameter. This shows the effects of a particular parameter regardless of all other parameters. For example, transmission and shedding rates have the strongest effects on mean daily prevalence, where higher rates mean higher prevalence. [file 1471-2334-13-592-S2.tiff]

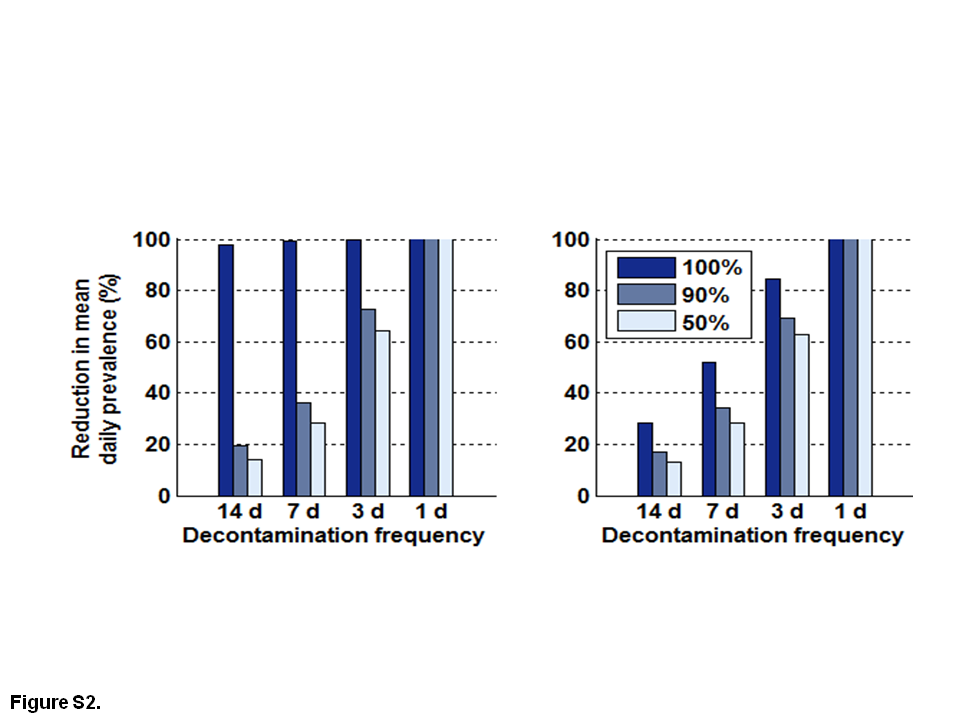

Supplement: Additional file 3: Figure S2 — Same as Figure 1B except that stay-time was 1 day. [file 1471-2334-13-592-S3.tiff]
